# *NOTCH1* and *CREBBP* co‐mutations negatively affect the benefit of adjuvant therapy in completely resected *EGFR*‐mutated NSCLC: translational research of phase III IMPACT study

**DOI:** 10.1002/1878-0261.13542

**Published:** 2023-10-28

**Authors:** Satoshi Ikeda, Masahiro Tsuboi, Kazuko Sakai, Toshihiro Misumi, Hiroaki Akamatsu, Hiroyasu Shoda, Noriaki Sakakura, Atsushi Nakamura, Yasuhisa Ohde, Hidetoshi Hayashi, Kyoichi Okishio, Morihito Okada, Ichiro Yoshino, Jiro Okami, Kazuhisa Takahashi, Norihiko Ikeda, Masayuki Tanahashi, Yuichi Tambo, Haruhiro Saito, Shinichi Toyooka, Hidetoshi Inokawa, Toyofumi Chen‐Yoshikawa, Toshihide Yokoyama, Tatsuro Okamoto, Noriko Yanagitani, Masahide Oki, Makoto Takahama, Kenji Sawa, Hirohito Tada, Kazuhiko Nakagawa, Tetsuya Mitsudomi, Kazuto Nishio

**Affiliations:** ^1^ Department of Respiratory Medicine Kanagawa Cardiovascular and Respiratory Center Yokohama Japan; ^2^ Division of Thoracic Surgery National Cancer Center Hospital East Kashiwa Japan; ^3^ Department of Genome Biology Kindai University Faculty of Medicine Osaka‐Sayama Japan; ^4^ Department of Data Science National Cancer Center Hospital East Kashiwa Japan; ^5^ Internal Medicine III Wakayama Medical University Japan; ^6^ Department of Respiratory Medicine Hiroshima Citizens Hospital Japan; ^7^ Department of Thoracic Surgery Aichi Cancer Center Hospital Nagoya Japan; ^8^ Department of Pulmonary Medicine Sendai Kousei Hospital Japan; ^9^ Division of Thoracic Surgery Shizuoka Cancer Center Sunto‐gun Japan; ^10^ Department of Medical Oncology Kindai University Faculty of Medicine Osaka‐Sayama Japan; ^11^ Department of Thoracic Oncology National Hospital Organization Kinki‐Chuo Chest Medical Center Sakai Japan; ^12^ Department of Surgical Oncology Hiroshima University Japan; ^13^ Department of General Thoracic Surgery Chiba University Graduate School of Medicine Japan; ^14^ Department of General Thoracic Surgery Osaka International Cancer Institute Japan; ^15^ Department of Respiratory Medicine Juntendo University Graduate School of Medicine Bunkyo‐ku Japan; ^16^ Department of Surgery Tokyo Medical University Shinjuku‐ku Japan; ^17^ Division of Thoracic Surgery, Respiratory Disease Center Seirei Mikatahara General Hospital Hamamatsu Japan; ^18^ Department of Respiratory Medicine Kanazawa University Hospital Japan; ^19^ Department of Thoracic Oncology Kanagawa Cancer Center Yokohama Japan; ^20^ Department of General Thoracic Surgery Okayama University Graduate School of Medicine Japan; ^21^ Department of Thoracic Surgery Yamaguchi‐Ube Medical Center Japan; ^22^ Department of Thoracic Surgery Nagoya University Graduate School of Medicine Japan; ^23^ Department of Respiratory Medicine Kurashiki Central Hospital Japan; ^24^ Department of Thoracic Oncology National Hospital Organization Kyushu Cancer Center Fukuoka Japan; ^25^ Department of Thoracic Medical Oncology Cancer Institute Hospital of Japanese Foundation for Cancer Research Koto‐ku Japan; ^26^ Department of Respiratory Medicine National Hospital Organization Nagoya Medical Center Japan; ^27^ Department of General Thoracic Surgery Osaka City General Hospital Japan; ^28^ Department of Clinical Oncology Osaka Metropolitan University Graduate School of Medicine Japan; ^29^ Department of Thoracic Surgery Suita Tokushukai Hospital Japan; ^30^ Division of Thoracic Surgery Kindai University Faculty of Medicine Osaka‐Sayama Japan

**Keywords:** adjuvant therapy, early‐stage, epidermal growth factor receptor, non‐small cell lung cancer, tyrosine kinase inhibitor

## Abstract

The phase III IMPACT study (UMIN000044738) compared adjuvant gefitinib with cisplatin plus vinorelbine (cis/vin) in completely resected *epidermal growth factor receptor* (*EGFR*)‐mutated non‐small cell lung cancer (NSCLC). Although the primary endpoint of disease‐free survival (DFS) was not met, we searched for molecular predictors of adjuvant gefitinib efficacy. Of 234 patients enrolled in the IMPACT study, 202 patients were analyzed for 409 cancer‐related gene mutations and tumor mutation burden using resected lung cancer specimens. Frequent somatic mutations included *tumor protein p53* (*TP53*; 58.4%), *CUB and Sushi multiple domains 3* (*CSMD3*; 11.8%), and *NOTCH1* (9.9%). Multivariate analysis showed that *NOTCH1* co‐mutation was a significant poor prognostic factor for overall survival (OS) in the gefitinib group and *cAMP response element binding protein* (*CREBBP*) co‐mutation for DFS and OS in the cis/vin group. In patients with *NOTCH1* co‐mutations, gefitinib group had a shorter OS than cis/vin group (Hazard ratio 5.49, 95% CI 1.07–28.00), with a significant interaction (*P* for interaction = 0.039). In patients with *CREBBP* co‐mutations, the gefitinib group had a longer DFS than the cis/vin group, with a significant interaction (*P* for interaction = 0.058). In completely resected *EGFR*‐mutated NSCLC, *NOTCH1* and *CREBBP* mutations might predict poor outcome in patients treated with gefitinib and cis/vin, respectively.

## Graphical abstract

This study explored biomarkers of adjuvant therapy for resected *epidermal growth factor receptor* (*EGFR*)‐mutated non‐small cell lung cancer using tumor specimens from the phase III IMPACT study. *NOTCH1* co‐mutation can serve as a poor prognostic factor for overall survival in the gefitinib group while probably predicting a poor response to adjuvant EGFR‐TKI. Meanwhile, *CREBBP* co‐mutation may be a biomarker for predicting a poor response to adjuvant platinum‐based chemotherapy.

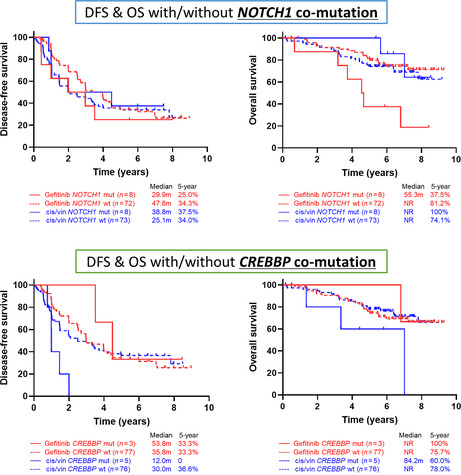

**Abbreviations**
cis/vin: cisplatin plus vinorelbine
CREBBP: cAMP response element binding protein
CSMD3: CUB and Sushi multiple domains 3
DCC: deleted in colorectal cancer
DFS: disease‐free survival
EGFR: epidermal growth factor receptor
FFPE: formalin‐fixed, paraffin‐embedded
HR: hazard ratio
KMT: lysine (K)‐specific methyltransferase
NGS: next‐generation sequencing
NSCLC: non‐small cell lung cancer
OS: overall survival
PPS: per protocol set
SYNE1: spectrin repeat containing nuclear envelope protein 1
TKI: tyrosine kinase inhibitor
TMB: tumor mutation burden
TP53: tumor protein p53

## 1 Introduction

Although patients who underwent complete resection of non‐small cell lung cancer (NSCLC) are expected to have favorable survival outcome, 5‐year disease‐free survival (DFS) and overall survival (OS) for completely resected NSCLC were low: 46–50% and 56–64% for pathological stage IIA‐IIB, and 28% and 48% for stage IIIA, respectively [1]. Since the recurrence often occurs as distant metastases, cisplatin‐based adjuvant chemotherapy has long been used as a standard‐of‐care. However, an increase in 5‐year survival rates is only 5–10% [2, 3].

For advanced NSCLC harboring *epidermal growth factor receptor* (*EGFR*) mutations, EGFR‐tyrosine kinase inhibitors (TKIs) have demonstrated higher efficacy than platinum‐based chemotherapy and are now the standard of care for the first‐line therapy [4, 5, 6, 7, 8, 9]. Thus, attempting to apply EGFR‐TKIs as adjuvant therapy after complete resection of *EGFR*‐mutated NSCLC is logical. The ADJUVANT/CTONG1104 study compared gefitinib with cisplatin/vinorelbine (cis/vin) for stage II–III *EGFR*‐mutated NSCLC and showed a significant prolongation of DFS by gefitinib (hazard ratio [HR] 0.56, *P* = 0.001) but not OS [10, 11]. We recently reported that, in a similarly designed WJOG6410L/IMPACT study, gefitinib failed to significantly prolong DFS and OS [12]. However, DFS curves of these two studies showed a similar trend: the gefitinib group was superior during the first 4 years, then the two curves crossed, and this initial superiority was not translated into an OS benefit. Meanwhile, the placebo‐controlled randomized phase III ADAURA study, launched later than the IMPACT and ADJUVANT/CTONG1104 trials, demonstrated that osimertinib for 3 years provided statistically significant and clinically meaningful DFS and OS benefits, as well as a reduction in central nervous system recurrence [13, 14]. Based on these results, osimertinib has been approved by various national and regional regulatory agencies. However, the 4‐year DFS rate of adjuvant osimertinib for *EGFR*‐mutated stage II–IIIA NSCLC is only 70%, and the presence of common *EGFR* mutations alone is still not a sufficient biomarker for adjuvant EGFR‐TKI.

To date, in advanced *EGFR*‐mutated NSCLC, co‐occurring mutations and/or amplifications in the *tumor protein p53* (*TP53*), *HER2*, *MET*, *CDK4/6* gene, etc., have been reported to negatively affect the survival outcome of the patients treated with EGFR‐TKIs [15, 16, 17, 18, 19, 20, 21, 22, 23]. We hypothesized that in the adjuvant setting, the benefits of gefitinib might be clinically meaningful by selecting patients who have specific molecular biomarkers among those with *EGFR* mutations. With these backgrounds, we decided to search for molecular predictors of efficacy using tumor specimens from the phase III IMPACT study.

## 2 Materials and methods

### 2.1 Clinical specimens

The eligibility criteria of this IMPACT‐TR study included (a) enrollment in the IMPACT study and (b) availability of surgically resected lung cancer tissue specimens. The registration period was from April 2021 to June 2022. Formalin‐fixed, paraffin‐embedded (FFPE) specimens of surgically resected tumors (10 slides of 4–5 μm thickness) were available in 211 of the 234 patients enrolled in the IMPACT study. These specimens were subjected to histologic examination and those that contained sufficient tumor cells (at least 10%) were used for nucleic acid extraction. DNA was isolated from tissues using the GeneRead DNA FFPE Kit (Qiagen, Valencia, CA, USA). The quality and quantity of nucleic acids were verified using a NanoDrop 2000 instrument and PicoGreen dsDNA Reagent (Thermo Scientific, Wilmington, DE, USA).

### 2.2 Next‐generation sequencing (NGS)

Co‐existing somatic mutation profiling and tumor mutation burden (TMB) estimation were performed using the Oncomine Tumor Mutation Load Assay (Thermo Fisher Scientific, Wilmington, DE, USA) in accordance with the manufacturer's recommended protocol. The assay covers 1.65 Mb across 409 oncogenes relevant across major cancer types (full list of the genes is available at https://www.thermofisher.com/order/catalog/product/A37909). Sequencing was performed using IonS5™ XL platform (Thermo Fisher Scientific). Sequencing data were analyzed with Ion Torrent Suite and Ion Reporter (Thermo Fisher Scientific). In order to evaluate the quality of the sequencing, on‐target alignment rate > 90%, mean depth > 500, and deamination ≤ 10 were set as the reference values. Samples that deviated from the reference values for any of the items were considered unassessable and subsequently excluded from further investigations in this study.

### 2.3 Ethics approval and consent to participate

This study was conducted in accordance with the Declaration of Helsinki. This study was approved by the Ethics Review Board of National Cancer Center Hospital East on February 22, 2021 (approval number: 2020‐401), and the Institutional Review Board or Ethics Committee of 23 participating facilities in Japan. This study was registered in the University Hospital Medical Information Network Clinical Trials Registry on July 2, 2021 (registry number: UMIN000044738). Written informed consent was obtained for patients who were still being followed up. Patients who had died or were no longer being followed up were opted out according to the criteria of each participating facilities.

### 2.4 Statistical analysis

Of the patients enrolled in this IMPACT‐TR study, those who fell within the Per Protocol Set (PPS) population as defined in the WJOG6410L/IMPACT study (excluding patients not receiving protocol treatment and ineligible patients from the ITT population) and successfully performed each of the measures were analyzed.

To explore whether co‐existing somatic mutations and TMB are associated with DFS and OS in completely resected *EGFR*‐mutated NSCLC who received adjuvant gefitinib, the following analyses were performed in the gefitinib group; (a) The detected co‐existing somatic mutations with a prevalence of ≥ 5% were considered as candidate biomarkers. TMB was categorized into TMB high and TMB low groups, with the median value as a cut‐off. (b) Univariate and multivariate analyses using the Cox proportional hazards model were performed to analyze the correlation between individual parameters and DFS or OS, and HRs and their 95% confidence intervals were calculated. Kaplan–Meier curves were used to estimate survival, and the log‐rank test was used to compare DFS and OS between groups. The same analyses described above were also performed in the cis/vin group.

Subsequently, we compared DFS and OS between the gefitinib and cis/vin groups among patient subgroups according to the biomarkers. The interaction of each biomarker was tested using the Cox proportional hazards model and *P* for interaction was calculated, with a *P*‐value of < 0.1 indicating a significant interaction between treatment and marker. Because *P* values for interactions have low statistical power and are unlikely to reach statistical significance, 0.1 or 0.2 is generally used as the significance level [24].

When comparing between groups, Fisher's exact test was used for nominal variables. The statistical significance level for all tests (excluding tests of interaction for each biomarker) was set at 5% (*P* = 0.05). jmp (ver. 14.0; SAS Institute Inc., Cary, NC, USA) and graphpad prism software (ver. 8; GraphPad Software Inc., San Diego, CA, USA) were used for the statistical analysis.

## 3 Results

### 3.1 Sample availability and clinical characteristics

**Fig. 1:**
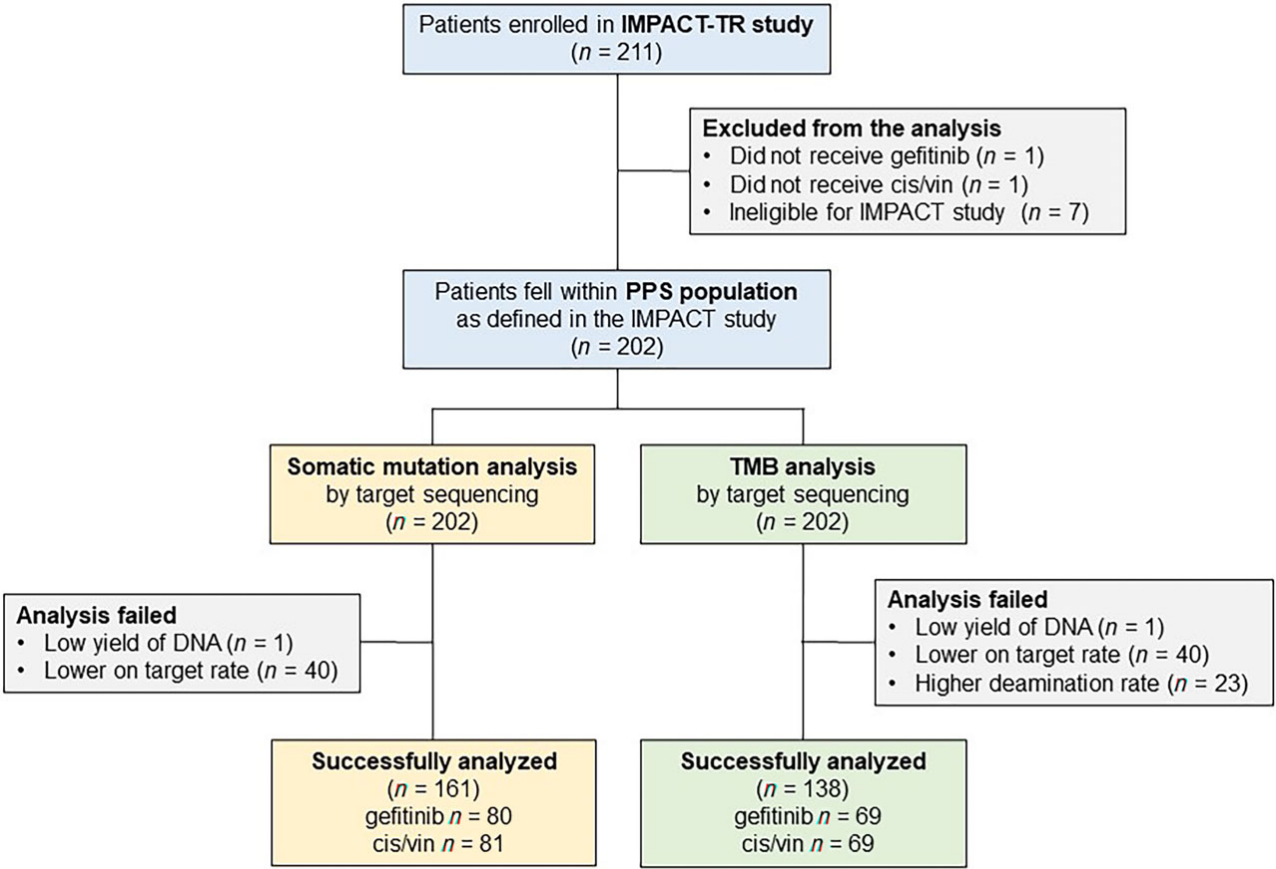
Patient disposition. Of the collected 211 specimens, 202 from patients who fell within the PPS population were analyzed. The success rates of somatic mutation analysis and TMB analysis were 79.7% and 68.3%, respectively.

Of the collected 211 specimens, 202 from patients who fell within the PPS population were analyzed (Fig. 1). Of these 202 patients, there were no differences in baseline patient characteristics, 5‐year DFS, or 5‐year OS between the gefitinib group (*N* = 101) and the cis/vin group (*N* = 101) (Table 1). The success rates of somatic mutation analysis and TMB analysis were 79.7% and 68.3%, respectively. Significant differences were not observed in patient characteristics among those successfully analyzed by somatic mutation analysis (*N* = 161) and TMB analysis (*N* = 138) (Table 1).

**Table 1:** Characteristics of patients successfully analyzed in each test. ECOG, Eastern Cooperative Oncology Group; PS, performance status.

	All analyzed patients (*N* = 202)				Successfully analyzed for co‐existing somatic mutations (*N* = 161)				Successfully analyzed for tumor mutation burden (*N* = 138)			
	Gefitinib group (*N* = 101)		Cis/vin group (*N* = 101)		Gefitinib group (*N* = 80)		Cis/vin group (*N* = 81)		Gefitinib group (*N* = 69)		Cis/vin group (*N* = 69)	
	*N*	%	*N*	%	*N*	%	*N*	%	*N*	%	*N*	%
Age												
< 65	54	53.5	50	49.5	39	48.8	39	48.1	33	47.8	33	47.8
≥ 65	47	46.5	51	50.5	41	51.3	42	51.9	36	52.2	36	52.2
Gender												
M	40	39.6	39	38.6	32	40.0	32	39.5	27	39.1	28	40.6
F	61	60.4	62	61.4	48	60.0	49	60.5	42	60.9	41	59.4
Stage												
IIA	32	31.7	34	33.7	27	33.8	32	39.5	20	29.0	30	43.5
IIB	5	5.0	3	3.0	4	5.0	3	3.7	4	5.8	1	1.4
IIIA	61	60.4	62	61.4	46	57.5	44	54.3	42	60.9	37	53.6
IIIB	3	3.0	2	2.0	3	3.8	2	2.5	3	4.3	1	1.4
ECOG PS												
0	83	82.2	78	77.2	66	82.5	60	74.1	56	81.2	50	72.5
1	18	17.8	23	22.8	14	17.5	21	25.9	13	18.8	19	27.5
Smoking history												
Former	41	40.6	36	35.6	34	42.5	28	34.6	28	40.6	25	36.2
Never	60	59.4	65	64.4	46	57.5	53	65.4	41	59.4	44	63.8
EGFR												
19del	57	56.4	51	50.0	47	58.8	40	48.8	42	60.9	37	52.9
L858R	44	43.6	51	50.0	33	41.3	42	51.2	27	39.1	33	47.1
	%	95% CI	%	95% CI	%	95% CI	%	95% CI	%	95% CI	%	95% CI
5‐Year DFS	32.3	23.8–42.1	31.4	23.0–41.3	33.3	23.8–44.5	34.3	24.6–45.5	32.8	22.7–44.9	36.1	25.4–48.3
5‐Year OS	77.6	68.3–84.7	75.2	65.6–82.8	76.6	65.9–84.8	76.8	66.1–84.9	78.8	67.3–87.0	75.7	63.9–84.5

### 3.2 Profile of NGS

**Fig. 2:**
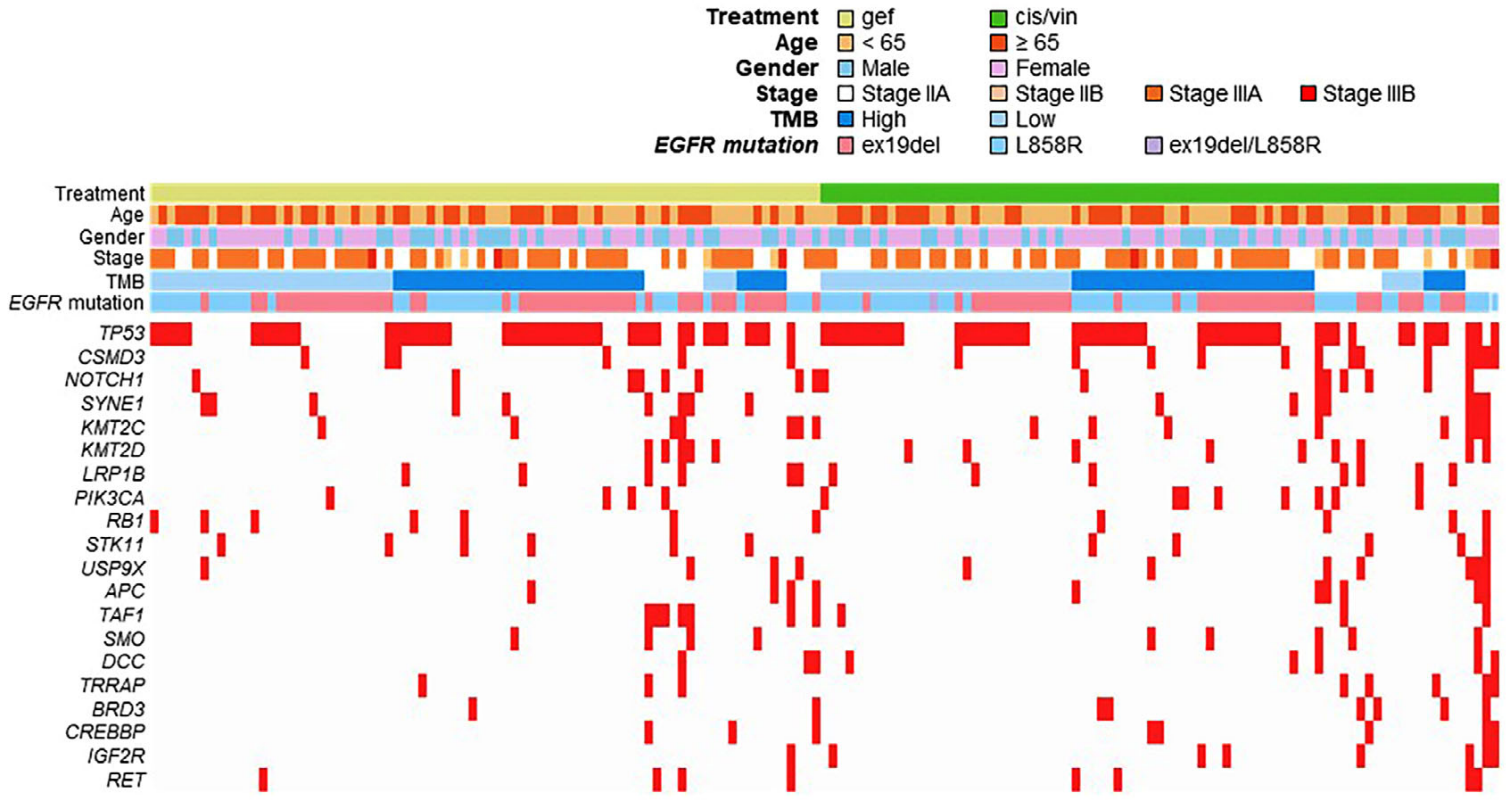
Profile of next‐generation sequencing. The detected co‐existing somatic mutations with a prevalence of ≥ 5% and the TMB status. APC, adenomatous polyposis coli; BRD3, bromodomain containing 3; IGF2R, insulin like growth factor 2 receptor; LRP1B, LDL receptor related protein 1B; PIK3CA, phosphatidylinositol‐4,5‐bisphosphate 3‐kinase catalytic subunit alpha; RB1, RB transcriptional corepressor 1; RET, ret proto‐oncogene; SMO, smoothened, frizzled class receptor; STK11, serine/threonine kinase 11; TAF1, TATA‐Box binding protein associated factor 1; TRRAP, transformation/transcription domain‐associated protein; USP9X, ubiquitin specific peptidase 9 X‐linked.

Co‐existing somatic mutations with a prevalence of ≥ 5% and the TMB values are shown in Fig. 2 and Table S1. The most frequent co‐existing somatic mutation was *TP53* (58.4%), followed by *CUB and Sushi multiple domains 3* (*CSMD3*, 11.8%), *NOTCH1* (9.9%) and *Spectrin Repeat Containing Nuclear Envelope Protein 1* (*SYNE1*, 9.9%). Co‐mutation of *cAMP response element binding protein* (*CREBBP*) was found in 5%. The median TMB was 6.67 mutations·Mb^−1^. There were no differences in the frequencies of various coexisting somatic mutations or TMB between the gefitinib and cis/vin group (Table S1).

### 3.3 Correlation of individual parameters with clinical outcomes

**Fig. 3:**
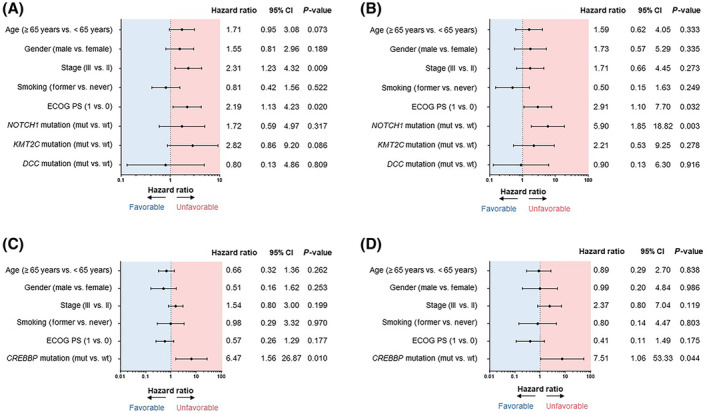
Multivariate analysis using Cox proportional hazards model. Univariate and multivariate analyses using the Cox proportional hazards model were performed to analyze the correlation between individual parameters and DFS/OS, and HRs and their 95% confidence intervals were calculated. Forest plots represent the results of multivariate analysis using the Cox proportional hazards model for each treatment group. DFS (A) and OS (B) in the gefitinib group; DFS (C) and OS (D) in the cis/vin group. ECOG, Eastern Cooperative Oncology Group; PS, performance status.

In the gefitinib group, univariate and multivariate analyses revealed no statistically significant predictors of DFS. However, *NOTCH1* mutation (HR 4.18, 95% CI 1.65–10.61, *P* = 0.003), *Lysine (K)‐specific methyltransferase (KMT) 2C* mutation (HR 2.91, 95% CI 1.09–7.82, *P* = 0.034) and *deleted in colorectal cancer* (*DCC*) mutation (HR 3.72, 95% CI 1.11–12.50, *P* = 0.034) were associated with shorter OS (Table S2). In multivariate analysis, no statistically significant predictors of DFS were found (Fig. 3A), but *NOTCH1* mutation (HR 5.90, 95% CI 1.85–18.82, *P* = 0.003) was associated with shorter OS (Fig. 3B). Figure S1 shows the Kaplan–Meier curves for DFS and OS compared with and without *NOTCH1* mutation in each treatment arms.

In the cis/vin group, multivariate analysis showed that *CREBBP* mutation was significantly associated with shorter DFS (HR 6.47, 95% CI 1.56–26.87, *P* = 0.010) and shorter OS (HR 7.51, 95% CI 1.06–53.33, *P* = 0.044) (Table S2, Fig. 3C,D). Figure S2 shows the Kaplan–Meier curves for DFS and OS compared with and without *CREBBP* mutation in each treatment arms.

*TP53* co‐mutation and TMB were not associated with DFS or OS in either treatment group.

### 3.4 Interaction between molecular biomarkers and treatment effect on DFS and OS

**Fig. 4:**
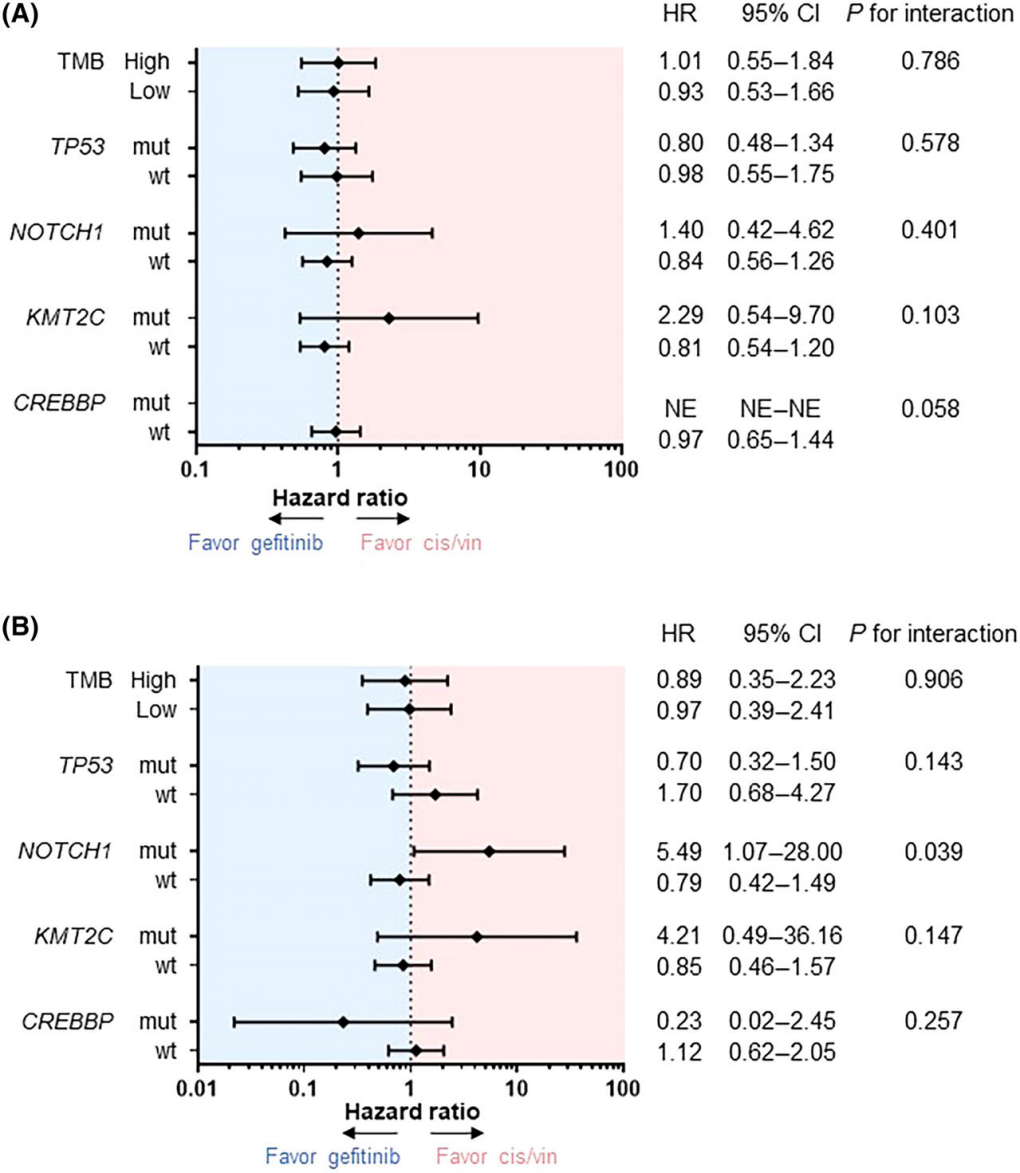
Subgroup analysis and interaction test. Forest plots showing comparison of DFS (A) and OS (B) between gefitinib and cis/vin groups in patient subgroups according to representative biomarkers, and results of interaction tests. The interaction of each biomarker was tested using the Cox proportional hazards model and *P* for interaction was calculated, with a *P*‐value of < 0.1 indicating a possible interaction between treatment and marker. NE, not evaluable.

The interaction of DFS and OS by treatment groups and biomarkers are shown in Fig. 4 and Table S3. Candidate biomarkers were selected based on the results of univariate and multivariate analyses described in the previous section (Fig. 3 and Table S2). In addition, *TP53* and TMB, which had been considered promising from previous reports and focused on in this study, were also added as candidate biomarkers. For the interaction test in this study, a *P*‐value of < 0.1 was considered to indicate an interaction between treatment and marker.

**Fig. 5:**
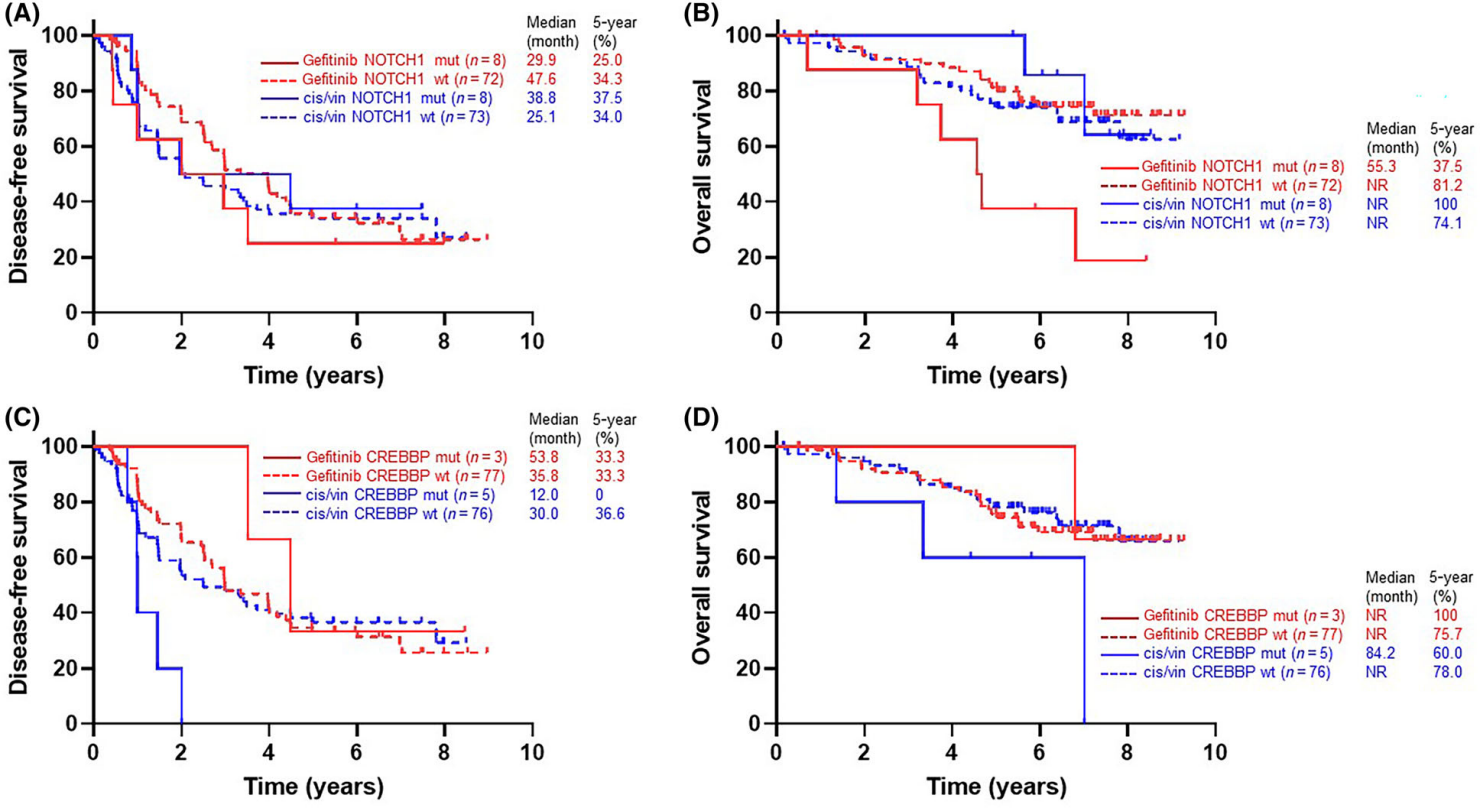
Kaplan–Meier curve with and without *NOTCH1* and *CERBBP* mutations. Kaplan–Meier curves for DFS (A) and OS (B), divided into four groups according to treatment (gefitinib [red] or cis/vin [blue]) and with [solid line] or without [dashed line] *NOTCH1* mutation. For CREBBP mutation, Kaplan–Meier curves for DFS (C) and OS (D) are also shown in the same way, divided into four groups. NR, not reached.

The interaction between *NOTCH1* co‐mutation and treatment effect on OS was significant (*P* for interaction = 0.039) (Fig. 4B). The gefitinib group had a shorter OS than the cis/vin group (median 55.3 months vs Not Reached; HR 5.49, 95% CI 1.07–28.00) in patients with *NOTCH1* mutation, but no such difference was observed in patients without *NOTCH1* mutation (Fig. 5B). The interaction between *NOTCH1* co‐mutation and DFS was not significant (Figs 4A and 5A).

The interaction between *CREBBP* co‐mutation and treatment effect on DFS was also significant (*P* for interaction = 0.058) (Fig. 4A). The gefitinib group had a longer DFS than the cis/vin group in patients with *CREBBP* mutation (median 53.8 months vs 12.0 months; HR Not Evaluable), but no such difference was observed in patients without *CREBBP* mutation (Fig. 5C). The interaction between *CREBBP* mutation and OS was not significant (Figs 4B and 5D).

## 4 Discussion

Few reports have investigated biomarkers predicting the efficacy of adjuvant therapy and the risk of postoperative recurrence in patients with completely resected *EGFR*‐mutated NSCLC, particularly in conjunction with data from interventional studies. The biomarker analysis of ADJUVANT/CTONG1104 study reported that the MINERVA score, which is calculated using five potential biomarkers of DFS (*RB1* alteration, *NKX2‐1* copy number gain, *CDK4* copy number gain, *TP53* exon4/5 missense alterations, and *MYC* copy number gain) identified by comprehensive genomic profiling, could be used to categorize resected *EGFR*‐mutated NSCLC into three subgroups by efficacy of adjuvant therapy (highly TKI‐preferable, TKI‐preferable, and chemotherapy‐preferable groups) [25]. However, due to the complexity of this scoring system and the small sample sizes, the results of the study did not provide a definitive biomarker.

This IMPACT‐TR study demonstrated two important findings. First, *NOTCH1* co‐mutation can serve as a poor prognostic factor for OS in the gefitinib group while probably predicting a poor response to adjuvant EGFR‐TKI. Second, *CREBBP* co‐mutation may be a biomarker for predicting a poor response to adjuvant platinum‐based chemotherapy for completely resected *EGFR*‐mutated NSCLC.

*NOTCH1* is a gene encoding a transmembrane protein that plays an important role in tumorigenesis, and *NOTCH1* mutations are frequently detected in colon adenocarcinoma, lung adenocarcinoma, and breast invasive ductal carcinoma [26]. *NOTCH1* has been reported to be associated with acquired resistance to gefitinib by triggering epithelial‐mesenchymal transition, both *in vivo* and *in vitro* [27, 28, 29]. Since there was a significant interaction between *NOTCH1* co‐mutation and treatment effect on OS in this IMPACT‐TR study, *NOTCH1* co‐mutation may predict the efficacy of adjuvant gefitinib rather than simply being a prognostic factor in postoperative *EGFR*‐mutated NSCLC. In fact, the biomarker analysis of the ADJUVANT/CTONG1104 study also demonstrated that *NOTCH1* co‐mutation tended to be associated with shorter DFS of gefitinib (HR 2.48, 95% CI 1.12–5.47), although it was detected less frequently at 5–6% [25]. The analysis of DFS did not show statistical significance in our study, but a trend was observed in the multivariate analysis of the gefitinib group, suggesting an association between *NOTCH1* co‐mutation and shorter DFS (HR 1.72, 95% CI 0.59–4.97, *P* = 0.317). A subgroup analysis of patients with *NOTCH1* co‐mutation also revealed a trend toward shorter DFS in the gefitinib group (HR 1.40, 95% CI 0.42–4.62, *P* for interaction = 0.401). Since the number of *NOTCH1* co‐mutation positive samples in both IMPACT‐TR and ADJUVANT/CTONG1104 studies was small, further studies exploring a larger number of cases are warranted. However, the 5‐year DFS rate with gefitinib in patients without *NOTCH1* co‐mutation is only 34.3% (Fig. 5), which is far below the data for adjuvant osimertinib in the ADAURA study. Therefore, it may be difficult to substitute gefitinib for osimertinib in the adjuvant setting, even in patients without *NOTCH1* co‐mutation. Furthermore, it is also necessary to verify whether *NOTCH1* co‐mutation can be a biomarker for osimertinib, the current standard for adjuvant EGFR‐TKI. As for the ADAURA study, data exploring biomarkers such as co‐mutaton are not yet available. The *NOTCH1* pathway has also been reported to be associated with osimertinib resistance, suggesting that *NOTCH1* co‐mutation could be a promising biomarker candidate [30]. To note, the frequency of *NOTCH1* co‐mutation in this study was skewed at 4.6% (4/87) for exon 19 deletion and 16.0% (12/75) for L858R. A subgroup analysis of the IMPACT study showed that patients with L858R had a poor response to adjuvant gefitinib, with a HR of 1.164 (95% CI 0.739–1.833) for DFS [12], which may have been partly due to this high frequency of *NOTCH1* co‐mutations. No such trend has been reported in previous reports, and confirmation in a larger number of cases is also warranted.

Meanwhile, *CREBBP* co‐mutation was not only significantly associated with shorter DFS and OS in multivariate analysis in the cis/vin group, but also showed a significant interaction in subgroup analysis of DFS in this study. These results suggest that *CREBBP* co‐mutation may be a predictor of adjuvant cis/vin efficacy for *EGFR*‐mutated NSCLC. *CREBBP* mutations are observed in various types of cancers including lung adenocarcinoma and are involved in carcinogenesis and progression [23]. In addition, it has been suggested that *CREBBP* mutation may lead to chemotherapy resistance via impaired histone acetylation and corresponding gene dysregulation, not only in hematologic tumors such as lymphoma and acute lymphoblastic leukemia [31, 32, 33], but also in various solid tumors such as breast cancer and small‐cell lung cancer [34, 35]. Although osimertinib is undoubtedly the key drug for postoperative adjuvant therapy in *EGFR*‐mutated NSCLC, platinum‐based chemotherapy prior to osimertinib remains the standard based on the ADAURA study design. However, the efficacy of adjuvant osimertinib is consistent with or without prior chemotherapy according to a subgroup analysis of the ADAURA study [29, 36]. Therefore, if there are significant toxicity concerns with adjuvant chemotherapy or the patient does not wish to receive it, the presence of *CREBBP* co‐mutation may support the decision to omit platinum‐based chemotherapy and start with osimertinib.

*TP53* co‐mutation can negatively affect the efficacy of EGFR‐TKI in advanced NSCLC and was one of the potential biomarkers found in the biomarker analysis of the ADJUVANT/CTONG1104 study. Although it was detected in 58.4% of the patients in this IMPACT‐TR study, no significant association with DFS or OS was found. There are several subtypes of *TP53* mutations, and the ADJUVANT/CTONG1104 study found only *TP53* exon4/5 missense alterations as potential biomarker for adjuvant gefitinib. Because the archival surgically resected specimens used in this IMPACT‐TR study are old, the *TP53* mutation subtypes might not have been accurately evaluated. Among the other potential biomarkers found in the ADJUVANT/CTONG1104 study, *RB1* mutation was also detected in 6.8% of the patients in this study, but showed no usefulness as a biomarker. Moreover, although higher TMB has been reported to be associated with better prognosis in patients with resected NSCLC [37] and was of interest in this study, no significant association with DFS/OS was demonstrated for *EGFR*‐mutated NSCLC in this study.

This study has some limitations. First, the success rates of NGS analysis were low (< 80%), probably attributed to the enrollment period for the IMPACT study (September 2011 to December 2015); this means that the archival surgically resected specimens used in this study were old. Second, the multiplicity of comparisons should be considered because analysis was performed on many co‐mutations for the number of enrolled patients. In this study, we initially planned to narrow down the candidate factors considering false discovery rate and to conduct a two‐step test by dividing into test and validation sets, but we abandoned the plan mainly because of the low frequency of each co‐mutation. Third, the number of patients with *NOTCH1* and *CREBBP* mutations in this study was small. Therefore, this study is only an exploratory biomarker study, and further validation using a larger number of patients is warranted.

## 5 Conclusion

In patients with completely resected *EGFR*‐mutated NSCLC, *NOTCH1* mutation was identified as a poor prognostic factor for OS in the gefitinib group and may serve as a predictor of a poor response to gefitinib. *CREBBP* mutation was identified as a poor prognostic factor for DFS and OS in the cis/vin group and may serve as a predictor of a poor response to cis/vin. Further studies in a larger number of patients, as well as validation of the utility of these biomarkers in patients receiving adjuvant osimertinib are warranted.

## Conflict of interest

Ikeda S received honoraria from AstraZeneca, Bristol Myers Squibb, Ono, Taiho, Chugai, Boehringer Ingelheim, Eli Lilly, Takeda, and Pfizer; research funding from AstraZeneca and Chugai; and took on a consulting or advisory roles for AstraZeneca, Chugai and Daiichi Sankyo. Tsuboi M received honoraria from AstraZeneca, Bristol Myers Squibb, Ono, Taiho, Chugai, Eli Lilly, Johnson & Johnson, Novartis, MSD, Medtronic and Daiichi Sankyo; research funding from AstraZeneca, Boehringer Ingelheim, Merck, Ono, Bristol Myers Squibb, Novartis, MiRXES Japan and BMG KK; and took on a consulting or advisory roles for AstraZeneca, Chugai, MSD and Novartis. Sakai K received honoraria from Chugai, Hitachi, Life Technologies, Takeda and Yodosha Co., Ltd. Misumi T has no relationships to disclose. Akamatsu H received honoraria from AstraZeneca, Eli Lilly, Pfizer, MSD, Bristol Myers Squibb, Ono, Taiho, Chugai, Boehringer Ingelheim, Amgen, Nippon Kayaku. Co. Ltd., Novartis and Takeda; research funding from Amgen and Chugai; and took on a consulting or advisory roles for Amgen, Janssen, and Sandoz K.K. Shoda H has no relationships to disclose. Sakakura N has no relationships to disclose. Nakamura A received honoraria from AstraZeneca, Chugai, Eli Lilly, Kyowa Kirin, Taiho, Novartis, Thermo Fisher, and MSD. Ohde Y has no relationships to disclose. Hayashi H received honoraria from AstraZeneca, Eli Lilly, Ono, Bristol Myers Squibb, Taiho, Boehringer Ingelheim, Chugai, Pfizer, MSD, Novartis and Merck Serono; research funding from MSD, Daiichi Sankyo, Taiho, Chugai, SYNEOS HEALTH CLINICAL, Japan Clinical Research Operations, AstraZeneca, IQVIA Services Japan, Covance Japan, Takeda, GlaxoSmithKline, Sanofi, EPS Corporation, Novartis, Medical Research Support, Bristol Myers Squibb, PRA Health Sciences, Janssen, Eisai, PAREXEL International, Ono, PPD‐SNBL, Nippon Boehringer Ingelheim, Sysmex, Eisai; and took on a consulting or advisory roles for Eli Lilly, AstraZeneca, Boehringer Ingelheim, Chugai, Pfizer, Merck Serono, Shanghai HaiHe Pharmaceutical, Bristol Myers Squibb and Daiichi Sankyo. Okishio K received honoraria from AstraZeneca, Bristol Myers Squibb, Chugai, Nippon Kayaku, Takeda, Taiho and Sawai Pharmaceutical. Okada M received honoraria from Taiho, Johnson & Johnson, Covidien, Eli Lilly, Chugai, AstraZeneca, Ono and CSL Behring; research funding from Taiho, Nippon Kayaku, Chugai, Covidien, Johnson & Johnson, Daiichi Sankyo, Yakult Honsha, Eli Lilly, Nihon Medi‐Physics, Pfizer, Mochida Pharmaceutical Co. Ltd., Shionogi, Ono and Kyowa Kirin. Yoshino I received honoraria from AstraZeneca, Chugai, Johnson & Johnson, Covidien, Taiho, Daiichi Sankyo, Japan Blood Products Organization, Bristol Myers Squibb, Shionogi, Eli Lilly, Astellas, CSL Behring, Ono, MSD and Care Net; research funding from Pfizer, Taiho, Shionogi and Daiichi Sankyo; and took on a consulting or advisory roles for AstraZeneca, Chugai, Johnson & Johnson, Medtronic and Medicaroid. Okami J has no relationships to disclose. Takahashi K received honoraria from AstraZeneca. Ikeda N received honoraria from AstraZeneca, Chugai, Boehringer Ingelheim, Taiho, Eli Lilly, Ono, Bristol Myers Squibb, Olympus, MSD, Johnson & Johnson, Medtronic and Teijin; research funding from AstraZeneca, Chugai, Boehringer Ingelheim, Pfizer, Taiho, Eli Lilly, Ono, Bristol Myers Squibb, MSD, Teijin, Sanofi, Astellas, Shionogi, Daiichi Sankyo, Roche, Fujifilm and Johnson & Johnson. Tanahashi M has no relationships to disclose. Tambo Y has no relationships to disclose. Saito H received honoraria from AstraZeneca and Ono; research funding from AstraZeneca, Chugai, Ono and Daiichi Sankyo. Toyooka S has no relationships to disclose. Inokawa H has no relationships to disclose. Chen‐Yoshikawa T has no relationships to disclose. Yokoyama T received honoraria from Takeda; research funding from Chugai, MSD, Takeda, Delta‐Fly Pharma and Janssen. Okamoto T received research funding from Chugai, MSD, Daiichi Sankyo, Pfizer and AnHeart. Yanagitani N received honoraria from Eli Lilly, Ono and Bristol Myers Squibb. Oki M received honoraria from AMCO, AstraZeneca, Canon Medical Systems Corporation, Chugai, Fujifilm Toyama Chemical, Kaneka Medix Corp, Merit Medical Japan, Novartis, Olympus, and Sanofi; research funding from AbbVie, AstraZeneca, Chugai, Fujifilm Toyama Chemical, GlaxoSmithKline, Janssen, MSD, Ono, Parexel, Pfizer, and Sanofi. Takahama M has no relationships to disclose. Sawa K received honoraria from Eli Lilly, AstraZeneca, Kyowa Kirin, Nippon Kayaku, Chugai, Taiho and ONO. Tada H received honoraria from Chugai. Nakagawa K received honoraria from AstraZeneca, Ono, Chugai, Boehringer Ingelheim, Taiho, Takeda, MSD, Merck, Bayer, Niopon ayaku, Amgen. Kyowa Kirin, Japan Clinical Research Operations, CMIC Co., Ltd., Taiyo Pharma, Eli Lilly, Pfizer, Novartis, CMIC, Life Technologies and Neo Communication; research funding from AstraZeneca, Chugai, Ono, Daiichi Sankyo, Boehringer Ingelheim, Taiho, PAREXEL, IQVIA, SymBio, Kissei, EPS holdings, Bayer, MSD, Otsuka, EPS International, PRA Health Science, PPD‐SNBL, Covance, GlaxoSmithKline, Sysmex, Mochida Pharmaceutical Co, Ltd, Sysmex, Japan Clinical Research Operations, Sanofi, Medical Research Support, Syneos Health, Nippon Kayaku, EPS holdings, Mebix, Janssen, Eli Lilly, Amgen, Novartis, SRL Diagnostics, Takeda, Eisai and Bristol‐Myers Squibb. Mitsudomi T received honoraria from AstraZeneca, Pfizer, Boehringer Ingelheim, Ono, Bristol Myers Squibb, Chugai, Taiho, Eli Lilly, Novartis, MSD, Kyowa Kirin, Amgen, Guardant Health, Ethicon, Thermofisher Scientific Biomarkers, Merck KGaA, Janssen and Takeda; research funding from AstraZeneca, Chugai, Boehringer Ingelheim, Pfizer, Taiho, Ono, MSD and Bridgebio; and took on a consulting or advisory roles for AstraZeneca, Ono, MSD and Amgen. Nishio K received honoraria from AstraZeneca, Chugai, Novartis, Otsuka, Merck Sharp & Dohme, Ono, Bristol‐Myers Squibb, SymBio Pharmaceuticals, Pfizer, Eli Lilly, Amgen, Merck, Yakult, Fujirebio, Gurdant Health, Takeda, Boehringer Ingelheim, Janssen, Daiichi Sankyo and Ono; research funding from Boehringer Ingelheim, Nichirei, Eli Lilly, Hitachi Cemical, SYSMEX and Otsuka.

## Author contributions

SI contributed to conceptualization, methodology, investigation, visualization, project administration, funding acquisition, and writing (original draft). MTsuboi contributed to conceptualization, supervision, project administration, funding acquisition, and writing (review and editing). KSakai contributed to methodology, formal analysis, visualization, and writing (review and editing). TMisumi contributed to methodology, formal analysis, data curation, and writing (review and editing). HA contributed to methodology, investigation, and writing (review and editing). HShoda, NS, AN, YO, HH, KO, MOkada, IY, JO, KT, NI, MTanahashi, YT, HSaito, ST, HI, TC‐Y, TY, TO, NY, MOki, MTakahama, KSawa, and HT contributed to investigation and writing (review and editing). KNakagawa contributed to investigation, supervision, and writing (review and editing). TMitsudomi contributed to methodology, investigation, and writing (original draft). KNishio contributed to methodology, formal analysis, supervision, and writing (review and editing).

### Peer review

The peer review history for this article is available at https://www.webofscience.com/api/gateway/wos/peer‐review/10.1002/1878‐0261.13542.

## Supporting information


**Fig. S1.** Kaplan–Meier curve of each treatment arms with and without *NOTCH1* mutation.

**Fig. S2.** Kaplan–Meier curve of each treatment arms with and without *CERBBP* mutation.

**Table S1.** Profile of next‐generation sequencing.
**Table S2.** Univariate analysis using Cox proportional hazards model.
**Table S3.** Subgroup analysis and interaction test.

## Data Availability

All data generated or analyzed during this study are included in this published article.

## References

[1] Okami J , Shintani Y , Okumura M , Ito H , Ohtsuka T , Toyooka S , et al. Demographics, safety and quality, and prognostic information in both the seventh and eighth editions of the TNM classification in 18,973 surgical cases of the Japanese Joint Committee of Lung Cancer Registry Database in 2010. J Thorac Oncol. 2019;14(2):212–222. 30316011 10.1016/j.jtho.2018.10.002

[2] Pignon JP , Tribodet H , Scagliotti GV , Douillard JY , Shepherd FA , Stephens RJ , et al. Lung adjuvant cisplatin evaluation: a pooled analysis by the LACE Collaborative Group. J Clin Oncol. 2008;26(21):3552–3559. 18506026 10.1200/JCO.2007.13.9030

[3] Douillard JY , Tribodet H , Aubert D , Shepherd FA , Rosell R , Ding K , et al. Adjuvant cisplatin and vinorelbine for completely resected non‐small cell lung cancer: subgroup analysis of the lung adjuvant cisplatin evaluation. J Thorac Oncol. 2010;5(2):220–228. 20027124 10.1097/JTO.0b013e3181c814e7

[4] Soria JC , Ohe Y , Vansteenkiste J , Reungwetwattana T , Chewaskulyong B , Lee KH , et al. Osimertinib in untreated EGFR‐mutated advanced non‐small‐cell lung cancer. N Engl J Med. 2018;378(2):113–125. 29151359 10.1056/NEJMoa1713137

[5] Hosomi Y , Morita S , Sugawara S , Kato T , Fukuhara T , Gemma A , et al. Gefitinib alone versus gefitinib plus chemotherapy for non‐small‐cell lung cancer with mutated epidermal growth factor receptor: NEJ009 Study. J Clin Oncol. 2020;38(2):115–123. 31682542 10.1200/JCO.19.01488

[6] Saito H , Fukuhara T , Furuya N , Watanabe K , Sugawara S , Iwasawa S , et al. Erlotinib plus bevacizumab versus erlotinib alone in patients with EGFR‐positive advanced non‐squamous non‐small‐cell lung cancer (NEJ026): interim analysis of an open‐label, randomised, multicentre, phase 3 trial. Lancet Oncol. 2019;20(5):625–635. 30975627 10.1016/S1470-2045(19)30035-X

[7] Nakagawa K , Garon EB , Seto T , Nishio M , Ponce Aix S , Paz‐Ares L , et al. Ramucirumab plus erlotinib in patients with untreated, EGFR‐mutated, advanced non‐small‐cell lung cancer (RELAY): a randomised, double‐blind, placebo‐controlled, phase 3 trial. Lancet Oncol. 2019;20(12):1655–1669. 31591063 10.1016/S1470-2045(19)30634-5

[8] Nishio K , Seto T , Nishio M , Reck M , Garon EB , Sakai K , et al. Ramucirumab plus erlotinib versus placebo plus erlotinib in patients with untreated metastatic EGFR‐mutated NSCLC: RELAY Japanese subset. JTO Clin Res Rep. 2021;2(6):100171. 34590023 10.1016/j.jtocrr.2021.100171 PMC8474372

[9] Wu YL , Cheng Y , Zhou X , Lee KH , Nakagawa K , Niho S , et al. Dacomitinib versus gefitinib as first‐line treatment for patients with EGFR‐mutation‐positive non‐small‐cell lung cancer (ARCHER 1050): a randomised, open‐label, phase 3 trial. Lancet Oncol. 2017;18(11):1454–1466. 28958502 10.1016/S1470-2045(17)30608-3

[10] Zhong WZ , Wang Q , Mao WM , Xu ST , Wu L , Shen Y , et al. Gefitinib versus vinorelbine plus cisplatin as adjuvant treatment for stage II‐IIIA (N1‐N2) EGFR‐mutant NSCLC (ADJUVANT/CTONG1104): a randomised, open‐label, phase 3 study. Lancet Oncol. 2018;19(1):139–148. 29174310 10.1016/S1470-2045(17)30729-5

[11] Zhong WZ , Wang Q , Mao WM , Xu ST , Wu L , Wei YC , et al. Gefitinib versus vinorelbine plus cisplatin as adjuvant treatment for stage II‐IIIA (N1‐N2) EGFR‐mutant NSCLC: final overall survival analysis of CTONG1104 phase III trial. J Clin Oncol. 2021;39(7):713–722. 33332190 10.1200/JCO.20.01820 PMC8078324

[12] Tada H , Mitsudomi T , Misumi T , Sugio K , Tsuboi M , Okamoto I , et al. Randomized phase III study of gefitinib versus cisplatin plus vinorelbine for patients with resected stage II‐IIIA non‐small‐cell lung cancer with EGFR mutation (IMPACT). J Clin Oncol. 2022;40(3):231–241. 34726958 10.1200/JCO.21.01729

[13] Wu YL , Tsuboi M , He J , John T , Grohe C , Majem M , et al. Osimertinib in resected EGFR‐mutated non‐small‐cell lung cancer. N Engl J Med. 2020;383(18):1711–1723. 32955177 10.1056/NEJMoa2027071

[14] Tsuboi M , Herbst RS , John T , Kato T , Majem M , Grohé C , et al. Overall survival with osimertinib in resected EGFR‐mutated NSCLC. N Engl J Med. 2023;389(2):137–147. 37272535 10.1056/NEJMoa2304594

[15] Kim Y , Lee B , Shim JH , Lee SH , Park WY , Choi YL , et al. Concurrent genetic alterations predict the progression to target therapy in EGFR‐mutated advanced NSCLC. J Thorac Oncol. 2019;14(2):193–202. 30391576 10.1016/j.jtho.2018.10.150

[16] Heredia D , Mas L , Cardona AF , Oyervides V , Motta Guerrero R , Galvez‐Nino M , et al. A high number of co‐occurring genomic alterations detected by NGS is associated with worse clinical outcomes in advanced EGFR‐mutant lung adenocarcinoma: data from LATAM population. Lung Cancer. 2022;174:133–140. 36379126 10.1016/j.lungcan.2022.11.002

[17] Nishio M , Paz‐Ares L , Reck M , Nakagawa K , Garon EB , Popat S , et al. RELAY, ramucirumab plus erlotinib (RAM+ERL) in untreated metastatic EGFR‐mutant NSCLC (EGFR+ NSCLC): association between TP53 status and clinical outcome. Clin Lung Cancer. 2023;24(5):415–428. 37076395 10.1016/j.cllc.2023.02.010

[18] Ohashi K , Ninomiya K , Yoshioka H , Bessho A , Shibayama T , Aoe K , et al. Impact of HER2 expression on EGFR‐TKI treatment outcomes in lung tumors harboring EGFR mutations: a HER2‐CS study subset analysis. Lung Cancer. 2020;150:83–89. 33096420 10.1016/j.lungcan.2020.09.024

[19] Nagasaka M , Singh V , Baca Y , Sukari A , Kim C , Mamdani H , et al. The effects of HER2 alterations in EGFR mutant non‐small cell lung cancer. Clin Lung Cancer. 2022;23(1):52–59. 34801409 10.1016/j.cllc.2021.08.012

[20] Benedettini E , Sholl LM , Peyton M , Reilly J , Ware C , Davis L , et al. Met activation in non‐small cell lung cancer is associated with de novo resistance to EGFR inhibitors and the development of brain metastasis. Am J Pathol. 2010;177(1):415–423. 20489150 10.2353/ajpath.2010.090863 PMC2893683

[21] Lai GGY , Lim TH , Lim J , Liew PJR , Kwang XL , Nahar R , et al. Clonal MET amplification as a determinant of tyrosine kinase inhibitor resistance in epidermal growth factor receptor‐mutant non‐small‐cell lung cancer. J Clin Oncol. 2019;37(11):876–884. 30676858 10.1200/JCO.18.00177

[22] Sitthideatphaiboon P , Teerapakpinyo C , Korphaisarn K , Leelayuwatanakul N , Pornpatrananrak N , Poungvarin N , et al. Co‐occurrence CDK4/6 amplification serves as biomarkers of de novo EGFR TKI resistance in sensitizing EGFR mutation non‐small cell lung cancer. Sci Rep. 2022;12(1):2167. 35140316 10.1038/s41598-022-06239-y PMC8828869

[23] Reis H , Metzenmacher M , Goetz M , Savvidou N , Darwiche K , Aigner C , et al. MET expression in advanced non‐small‐cell lung cancer: effect on clinical outcomes of chemotherapy, targeted therapy, and immunotherapy. Clin Lung Cancer. 2018;19(4):e441–e463. 29631966 10.1016/j.cllc.2018.03.010

[24] Rothman KJ , VanderWeele TJ , Lash TL , Haneuse S . Modern epidemiology. 4th ed. Philadelphia: Lippincott Williams & Wilkins; 2021.

[25] Liu SY , Bao H , Wang Q , Mao WM , Chen Y , Tong X , et al. Genomic signatures define three subtypes of EGFR‐mutant stage II‐III non‐small‐cell lung cancer with distinct adjuvant therapy outcomes. Nat Commun. 2021;12(1):6450. 10.1038/s41467-021-26806-7 PMC8575965 34750392

[26] AACR Project GENIE Consortium . AACR project GENIE: powering precision medicine through an international consortium. Cancer Discov. 2017;7(8):818–831. 28572459 10.1158/2159-8290.CD-17-0151 PMC5611790

[27] Xie M , He CS , Wei SH , Zhang L . Notch‐1 contributes to epidermal growth factor receptor tyrosine kinase inhibitor acquired resistance in non‐small cell lung cancer in vitro and in vivo. Eur J Cancer. 2013;49(16):3559–3572. 23916913 10.1016/j.ejca.2013.07.007

[28] Zhu X , Chen L , Liu L , Niu X . EMT‐mediated acquired EGFR‐TKI resistance in NSCLC: mechanisms and strategies. Front Oncol. 2019;9:1044. 31681582 10.3389/fonc.2019.01044 PMC6798878

[29] Iommelli F , De Rosa V , Terlizzi C , Fonti R , Camerlingo R , Stoppelli MP , et al. A reversible shift of driver dependence from EGFR to Notch1 in non‐small cell lung cancer as a cause of resistance to tyrosine kinase inhibitors. Cancers (Basel). 2021;13(9):2022. 33922104 10.3390/cancers13092022 PMC8122511

[30] Takahashi H , Sakakibara‐Konishi J , Furuta M , Shoji T , Tsuji K , Morinaga D , et al. Notch pathway regulates osimertinib drug‐tolerant persistence in EGFR‐mutated non‐small‐cell lung cancer. Cancer Sci. 2023;114(4):1635–1650. 36411521 10.1111/cas.15674 PMC10067397

[31] Mullighan CG , Zhang J , Kasper LH , Lerach S , Payne‐Turner D , Phillips LA , et al. CREBBP mutations in relapsed acute lymphoblastic leukaemia. Nature. 2011;471(7337):235–239. 21390130 10.1038/nature09727 PMC3076610

[32] Gao C , Liu SG , Lu WT , Yue ZX , Zhao XX , Xing TY , et al. Downregulating CREBBP inhibits proliferation and cell cycle progression and induces daunorubicin resistance in leukemia cells. Mol Med Rep. 2020;22(4):2905–2915. 32945392 10.3892/mmr.2020.11347 PMC7453649

[33] Zhou XR , Li X , Liao LP , Han J , Huang J , Li JC , et al. P300/CBP inhibition sensitizes mantle cell lymphoma to PI3Kδ inhibitor idelalisib. Acta Pharmacol Sin. 2022;43(2):457–469. 33850273 10.1038/s41401-021-00643-2 PMC8791947

[34] Razavi P , Chang MT , Xu G , Bandlamudi C , Ross DS , Vasan N , et al. The genomic landscape of endocrine‐resistant advanced breast cancers. Cancer Cell. 2018;34(3):427–438.e6. 30205045 10.1016/j.ccell.2018.08.008 PMC6327853

[35] George J , Lim JS , Jang SJ , Cun Y , Ozretić L , Kong G , et al. Comprehensive genomic profiles of small cell lung cancer. Nature. 2015;524(7563):47–53. 26168399 10.1038/nature14664 PMC4861069

[36] Wu YL , John T , Grohe C , Majem M , Goldman JW , Kim SW , et al. Postoperative chemotherapy use and outcomes from ADAURA: osimertinib as adjuvant therapy for resected EGFR‐mutated NSCLC. J Thorac Oncol. 2022;17(3):423–433. 34740861 10.1016/j.jtho.2021.10.014

[37] Devarakonda S , Rotolo F , Tsao MS , Lanc I , Brambilla E , Masood A , et al. Tumor mutation burden as a biomarker in resected non‐small‐cell lung cancer. J Clin Oncol. 2018;36(30):2995–3006. 30106638 10.1200/JCO.2018.78.1963 PMC6804865

