# Supplementary figures and images for: NOTCH1 and CREBBP co‐mutations negatively affect the benefit of adjuvant therapy in completely resected EGFR‐mutated NSCLC: translational research of phase III IMPACT study
Source: Mol Oncol. 2023 Oct 28;18(2):305–16. doi: 10.1002/1878-0261.13542 (PMC10850799; doi:10.1002/1878-0261.13542)

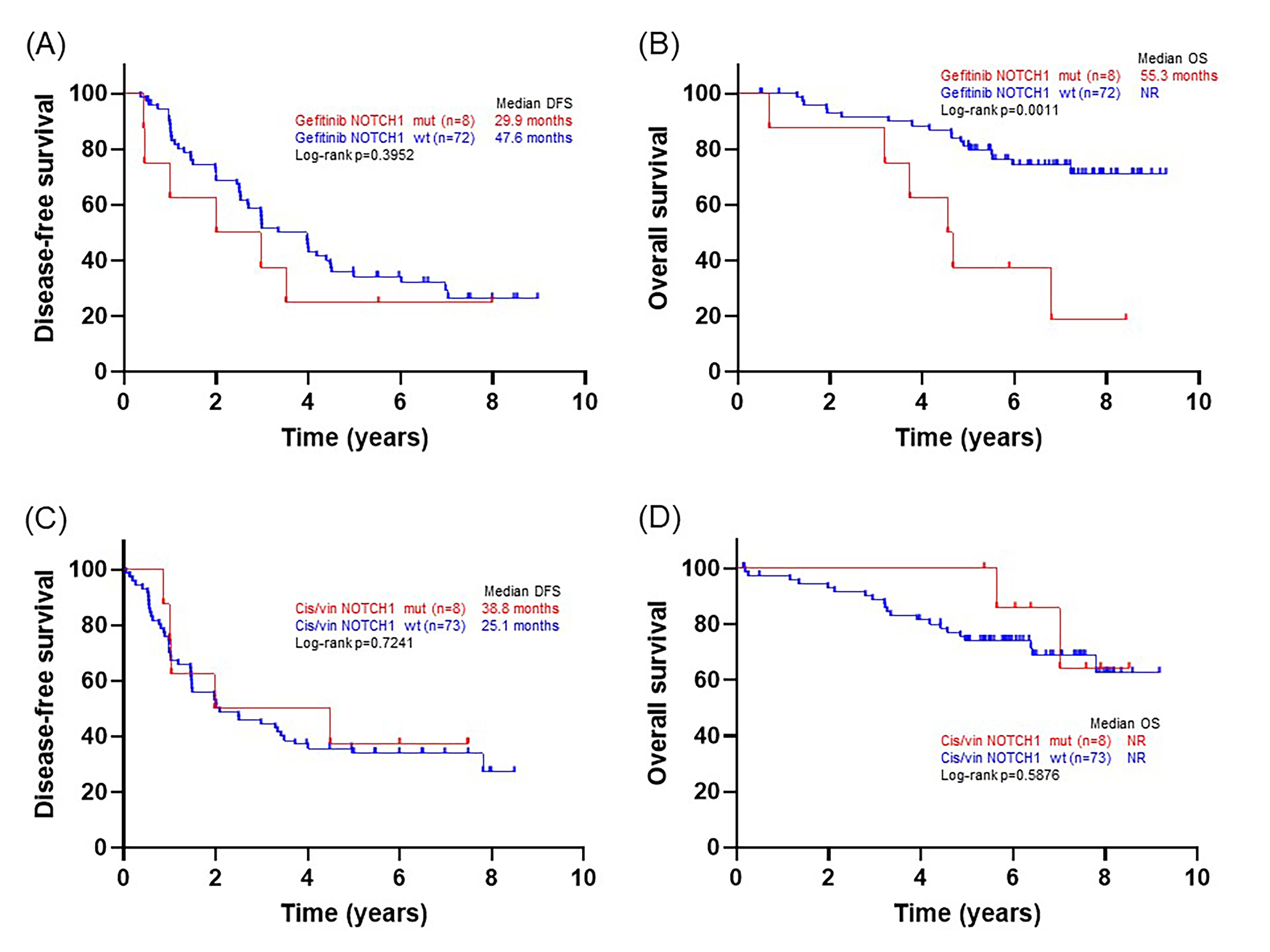

Supplement: Supplementary file 1 — Fig. S1. Kaplan–Meier curve of each treatment arms with and without NOTCH1 mutation. [file MOL2-18-305-s002.jpg]

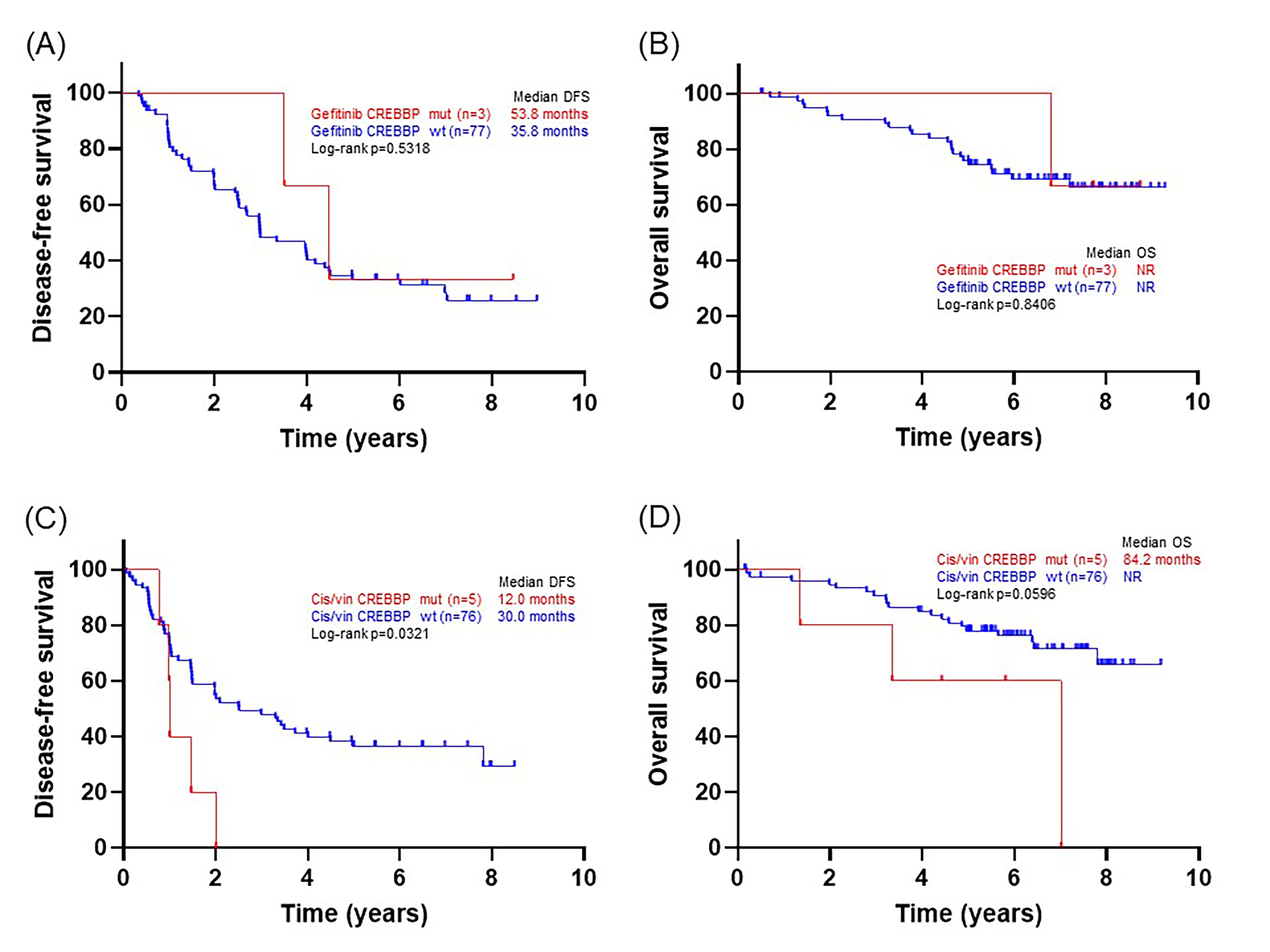

Supplement: Supplementary file 2 — Fig. S2. Kaplan–Meier curve of each treatment arms with and without CERBBP mutation. [file MOL2-18-305-s003.jpg]
