# Supplementary material for: NOTCH1 and CREBBP co‐mutations negatively affect the benefit of adjuvant therapy in completely resected EGFR‐mutated NSCLC: translational research of phase III IMPACT study
Source: Mol Oncol. 2023 Oct 28;18(2):305–16. doi: 10.1002/1878-0261.13542 (PMC10850799; doi:10.1002/1878-0261.13542)
Supplement: Supplementary file 3 — Table S1. Profile of next‐generation sequencing. Table S2. Univariate analysis using Cox proportional hazards model. Table S3. Subgroup analysis and interaction test. [file MOL2-18-305-s001.docx]

**Figure legends**

**Supplementary Figure 1.** **Kaplan-Meier curve of each treatment arms with and without *NOTCH1* mutation.**

The top two Figures show Kaplan-Meier curves of disease-free survival (A) and overall survival (B) in the gefitinib group compared with [red line] or without [blue line] *NOTCH1* mutation. The bottom two Figures show Kaplan-Meier curves of disease-free survival (C) and overall survival (D) in the cisplatin plus vinorelbine group compared with and without *NOTCH1* mutation [red line] or not [blue line]. Abbreviations: DFS, disease-free survival; OS, overall survival.

**Supplementary Figure 2. Kaplan-Meier curve of each treatment arms with and without *CERBBP* mutation.**

The top two Figures show Kaplan-Meier curves of disease-free survival (A) and overall survival (B) in the gefitinib group compared with [red line] or without [blue line] *CREBBP* mutation. The bottom two Figures show Kaplan-Meier curves of disease-free survival (C) and overall survival (D) in the cisplatin plus vinorelbine group compared with and without *CREBBP* mutation [red line] or not [blue line]. Abbreviations: DFS, disease-free survival; OS, overall survival; *CREBBP*, cAMP response element binding protein.

**Tables**

**Supplementary Table 1. Profile of next-generation sequencing**

|  | overall population | |  | gefitinib group | |  | cis/vin group | |  | p-value |
| --- | --- | --- | --- | --- | --- | --- | --- | --- | --- | --- |
|  | N | % |  | N | % |  | N | % |  |  |
| **Mutation profile (N=161)** |  |  |  |  |  |  |  |  |  |  |
| *TP53* | 94 | 58.4% |  | 44 | 55.0% |  | 50 | 61.7% |  | 0.426 |
| *CSMD3* | 19 | 11.8% |  | 6 | 7.5% |  | 13 | 16.0% |  | 0.141 |
| *NOTCH1* | 16 | 9.9% |  | 8 | 10.0% |  | 8 | 9.9% |  | 1.000 |
| *SYNE1* | 16 | 9.9% |  | 9 | 11.3% |  | 7 | 8.6% |  | 0.609 |
| *KMT2C* | 15 | 9.3% |  | 7 | 8.8% |  | 8 | 9.9% |  | 1.000 |
| *KMT2D* | 14 | 8.7% |  | 5 | 6.3% |  | 9 | 11.1% |  | 0.403 |
| *LRP1B* | 13 | 8.1% |  | 6 | 7.5% |  | 7 | 8.6% |  | 1.000 |
| *PIK3CA* | 12 | 7.5% |  | 4 | 5.0% |  | 8 | 9.9% |  | 0.369 |
| *RB1* | 11 | 6.8% |  | 7 | 8.8% |  | 4 | 4.9% |  | 0.369 |
| *STK11* | 11 | 6.8% |  | 6 | 7.5% |  | 5 | 6.2% |  | 0.766 |
| *USP9X* | 11 | 6.8% |  | 4 | 5.0% |  | 7 | 8.6% |  | 0.534 |
| *APC* | 10 | 6.2% |  | 4 | 5.0% |  | 6 | 7.4% |  | 0.746 |
| *TAF1* | 10 | 6.2% |  | 7 | 8.8% |  | 3 | 3.7% |  | 0.210 |
| *SMO* | 9 | 5.6% |  | 4 | 5.0% |  | 5 | 6.2% |  | 1.000 |
| *DCC* | 8 | 5.0% |  | 3 | 3.8% |  | 5 | 6.2% |  | 0.720 |
| *TRRAP* | 8 | 5.0% |  | 3 | 3.8% |  | 5 | 6.2% |  | 0.720 |
| *BRD3* | 8 | 5.0% |  | 2 | 2.5% |  | 6 | 7.4% |  | 0.277 |
| *CREBBP* | 8 | 5.0% |  | 3 | 3.8% |  | 5 | 6.2% |  | 0.720 |
| *IGF2R* | 8 | 5.0% |  | 1 | 1.3% |  | 7 | 8.6% |  | 0.064 |
| *RET* | 8 | 5.0% |  | 4 | 5.0% |  | 4 | 4.9% |  | 1.000 |
|  |  |  |  |  |  |  |  |  |  |  |
| **Tumor Mutation Burden (N=138)** | |  |  |  |  |  |  |  |  |  |
| High (cutoff value; median 6.67) | 70 | 50.7% |  | 36 | 52.2% |  | 34 | 49.3% |  | 0.865 |

List of co-existing somatic mutations with a prevalence of ≥5% and tumor mutation burden status. Fisher's exact test was used to compare the frequency in the gefitinib and cis/vin groups. Abbreviations: DFS, disease-free survival; OS, overall survival; cis/vin, cisplatin plus vinorelbine; ECOG, Eastern Cooperative Oncology Group; PS, Performance Status; TP53, Tumor Protein p53; CSMD3, CUB and Sushi multiple domains 3; SYNE1, Spectrin Repeat Containing Nuclear Envelope Protein 1; KMT, Lysine (K)-specific methyltransferase; LRP1B, LDL Receptor Related Protein 1B; PIK3CA, Phosphatidylinositol-4,5-bisphosphate 3-kinase catalytic subunit alpha; RB1, RB Transcriptional Corepressor 1; STK11, serine/threonine kinase 11; USP9X, Ubiquitin Specific Peptidase 9 X-Linked; APC, adenomatous polyposis coli; TAF1, TATA-Box Binding Protein Associated Factor 1; SMO, Smoothened, Frizzled Class Receptor; DCC, deleted in colorectal cancer; TRRAP, Transformation/Transcription Domain-Associated Protein; BRD3, Bromodomain Containing 3; CREBBP, cAMP response element binding protein; IGF2R, Insulin Like Growth Factor 2 Receptor; RET, ret proto-oncogene.

**Supplementary Table 2. Univariate analysis using Cox proportional hazards model**

|  |  |  | DFS in gefitinib arm | | | |  | OS in gefitinib arm | | | |  | DFS in cis/vin arm | | | |  | OS in cis/vin arm | | | |
| --- | --- | --- | --- | --- | --- | --- | --- | --- | --- | --- | --- | --- | --- | --- | --- | --- | --- | --- | --- | --- | --- |
|  |  |  | HR | 95%CI | | p |  | HR | 95%CI | | p |  | HR | 95%CI | | p |  | HR | 95%CI | | p |
|  |  |  |  | lower | upper |  |  |  | lower | upper |  |  |  | lower | upper |  |  |  | lower | upper |  |
| Age | >65 yrs vs. < 65 yrs |  | 1.02 | 0.64 | 1.62 | 0.929 |  | 1.01 | 0.50 | 2.03 | 0.976 |  | 1.23 | 0.76 | 1.98 | 0.403 |  | 1.94 | 0.94 | 4.03 | 0.074 |
| Gender | Male vs. Female |  | 1.15 | 0.72 | 1.84 | 0.562 |  | 1.16 | 0.58 | 2.33 | 0.680 |  | 0.71 | 0.43 | 1.17 | 0.179 |  | 1.01 | 0.49 | 2.09 | 0.970 |
| Stage | III vs. II |  | 1.76 | 1.06 | 2.91 | 0.029 |  | 2.00 | 0.90 | 4.46 | 0.089 |  | 1.77 | 1.04 | 3.01 | 0.035 |  | 1.70 | 0.76 | 3.81 | 0.196 |
| Smoking | former vs. never |  | 0.95 | 0.59 | 1.53 | 0.830 |  | 0.87 | 0.43 | 1.77 | 0.701 |  | 0.89 | 0.54 | 1.47 | 0.660 |  | 1.26 | 0.62 | 2.58 | 0.522 |
| ECOG PS | 1 vs. 0 |  | 1.76 | 1.01 | 3.08 | 0.047 |  | 2.02 | 0.94 | 4.38 | 0.073 |  | 0.81 | 0.45 | 1.43 | 0.465 |  | 0.62 | 0.24 | 1.62 | 0.331 |
| TMB (median) | high vs. low |  | 0.91 | 0.51 | 1.60 | 0.738 |  | 0.92 | 0.37 | 2.32 | 0.862 |  | 0.82 | 0.45 | 1.50 | 0.516 |  | 0.94 | 0.38 | 2.31 | 0.891 |
| *EGFR* mutation | ex19 del vs L858R |  | 0.80 | 0.47 | 1.37 | 0.418 |  | 0.56 | 0.25 | 1.28 | 0.168 |  | 0.74 | 0.42 | 1.31 | 0.298 |  | 0.81 | 0.36 | 1.86 | 0.626 |
| *TP53* | mut vs. wt |  | 0.71 | 0.41 | 1.20 | 0.200 |  | 0.61 | 0.27 | 1.36 | 0.224 |  | 0.94 | 0.54 | 1.65 | 0.841 |  | 1.50 | 0.61 | 3.64 | 0.375 |
| *CSMD3* | mut vs. wt |  | 2.30 | 0.97 | 5.48 | 0.060 |  | 0.86 | 0.20 | 3.64 | 0.833 |  | 0.93 | 0.44 | 1.99 | 0.857 |  | 0.52 | 0.12 | 2.22 | 0.377 |
| *NOTCH1* | mut vs. wt |  | 1.44 | 0.62 | 3.37 | 0.399 |  | 4.18 | 1.65 | 10.61 | **0.003*** |  | 0.85 | 0.34 | 2.13 | 0.725 |  | 0.67 | 0.16 | 2.87 | 0.590 |
| *SYNE1* | mut vs. wt |  | 0.83 | 0.36 | 1.95 | 0.672 |  | 0.60 | 0.14 | 2.56 | 0.490 |  | 1.20 | 0.47 | 3.01 | 0.705 |  | 1.64 | 0.49 | 5.55 | 0.425 |
| *KMT2C* | mut vs. wt |  | 2.20 | 0.93 | 5.18 | 0.073 |  | 2.91 | 1.09 | 7.82 | **0.034*** |  | 0.66 | 0.20 | 2.11 | 0.481 |  | 0.59 | 0.08 | 4.40 | 0.608 |
| *KMT2D* | mut vs. wt |  | 1.00 | 0.31 | 3.22 | 0.996 |  | 1.95 | 0.58 | 6.56 | 0.280 |  | 0.59 | 0.21 | 1.65 | 0.318 |  | 2.25 | 0.76 | 6.67 | 0.144 |
| *LRP1B* | mut vs. wt |  | 1.81 | 0.72 | 4.55 | 0.210 |  | 0.97 | 0.23 | 4.12 | 0.963 |  | 1.47 | 0.58 | 3.71 | 0.412 |  | 1.11 | 0.26 | 4.73 | 0.889 |
| *PIK3CA* | mut vs. wt |  | 1.43 | 0.44 | 4.59 | 0.550 |  | 0.73 | 0.10 | 5.39 | 0.755 |  | 1.28 | 0.51 | 3.22 | 0.603 |  | 0.45 | 0.06 | 3.37 | 0.439 |
| *RB1* | mut vs. wt |  | 0.40 | 0.12 | 1.28 | 0.121 |  | 0.36 | 0.05 | 2.66 | 0.317 |  | 1.74 | 0.54 | 5.63 | 0.354 |  | 1.64 | 0.38 | 7.08 | 0.506 |
| *STK11* | mut vs. wt |  | 0.57 | 0.18 | 1.82 | 0.341 |  | ND | ND | ND | - |  | 1.06 | 0.33 | 3.41 | 0.921 |  | 1.41 | 0.33 | 6.03 | 0.646 |
| *USP9X* | mut vs. wt |  | 1.82 | 0.56 | 5.88 | 0.317 |  | 2.41 | 0.56 | 10.30 | 0.235 |  | 1.83 | 0.73 | 4.62 | 0.200 |  | 1.85 | 0.55 | 6.28 | 0.320 |
| *APC* | mut vs. wt |  | 1.33 | 0.41 | 4.27 | 0.631 |  | 1.71 | 0.40 | 7.29 | 0.467 |  | 2.26 | 0.96 | 5.33 | 0.062 |  | 2.24 | 0.66 | 7.60 | 0.196 |
| *TAF1* | mut vs. wt |  | 1.30 | 0.51 | 3.26 | 0.583 |  | 1.95 | 0.66 | 5.73 | 0.225 |  | 1.09 | 0.26 | 4.47 | 0.909 |  | 1.71 | 0.23 | 12.80 | 0.603 |
| *SMO* | mut vs. wt |  | 0.24 | 0.03 | 1.71 | 0.153 |  | 0.71 | 0.10 | 5.27 | 0.738 |  | 0.86 | 0.27 | 2.75 | 0.793 |  | ND | ND | ND | - |
| *DCC* | mut vs. wt |  | 3.07 | 0.94 | 9.99 | 0.063 |  | 3.72 | 1.11 | 12.50 | **0.034*** |  | 1.19 | 0.43 | 3.29 | 0.744 |  | ND | ND | ND | - |
| *TRRAP* | mut vs. wt |  | 0.87 | 0.21 | 3.58 | 0.848 |  | 0.79 | 0.11 | 5.86 | 0.815 |  | 2.28 | 0.89 | 5.83 | 0.086 |  | 3.14 | 0.93 | 10.68 | 0.066 |
| *BRD3* | mut vs. wt |  | 0.54 | 0.07 | 3.91 | 0.542 |  | 1.54 | 0.21 | 11.43 | 0.673 |  | 0.53 | 0.13 | 2.17 | 0.374 |  | 0.74 | 0.10 | 5.51 | 0.770 |
| *CREBBP* | mut vs. wt |  | 0.64 | 0.16 | 2.63 | 0.536 |  | 0.81 | 0.11 | 6.05 | 0.841 |  | 2.70 | 1.05 | 6.97 | **0.040*** |  | 3.05 | 0.90 | 10.37 | 0.074 |
| *IGF2R* | mut vs. wt |  | 3.56 | 0.48 | 26.58 | 0.216 |  | ND | ND | ND | - |  | 0.99 | 0.36 | 2.76 | 0.990 |  | 2.70 | 0.79 | 9.17 | 0.113 |
| *RET* | mut vs. wt |  | 1.65 | 0.51 | 5.33 | 0.400 |  | 1.58 | 0.37 | 6.77 | 0.536 |  | 1.11 | 0.35 | 3.58 | 0.859 |  | 0.73 | 0.10 | 5.44 | 0.758 |

The results of univariate analysis using the Cox proportional hazards model to analyze the correlation between individual parameters and DFS or OS in each treatment group. Hazard ratios and their 95% confidence intervals were calculated, and a p-value <0.05 was considered statistically significant (*). Abbreviations: cis/vin, cisplatin plus vinorelbine; HR, Hazard ratio;TP53, Tumor Protein p53; CSMD3, CUB and Sushi multiple domains 3; SYNE1, Spectrin Repeat Containing Nuclear Envelope Protein 1; KMT, Lysine (K)-specific methyltransferase; LRP1B, LDL Receptor Related Protein 1B; PIK3CA, Phosphatidylinositol-4,5-bisphosphate 3-kinase catalytic subunit alpha; RB1, RB Transcriptional Corepressor 1; STK11, serine/threonine kinase 11; USP9X, Ubiquitin Specific Peptidase 9 X-Linked; APC, adenomatous polyposis coli; TAF1, TATA-Box Binding Protein Associated Factor 1; SMO, Smoothened, Frizzled Class Receptor; DCC, deleted in colorectal cancer; TRRAP, Transformation/Transcription Domain-Associated Protein; BRD3, Bromodomain Containing 3; CREBBP, cAMP response element binding protein; IGF2R, Insulin Like Growth Factor 2 Receptor; RET, ret proto-oncogene.

**Supplementary Table 3. Subgroup analysis and interaction test**

|  |  | Disease Free Survival | | | |  | Overall Survival | | | |
| --- | --- | --- | --- | --- | --- | --- | --- | --- | --- | --- |
|  |  | Hazard ratio | 95%CI | | P for interaction |  | Hazard ratio | 95%CI | | P for interaction |
|  |  |  | lower | upper |  |  |  | lower | upper |  |
| Age | <65 | 0.95 | 1.53 | 0.84 | 0.499 |  | 1.40 | 2.94 | 0.37 | 0.194 |
|  | ≥65 | 0.76 | 1.22 | 0.26 |  |  | 0.72 | 1.42 | 0.34 |  |
| Sex | male | 1.18 | 2.03 | 0.55 | 0.136 |  | 1.07 | 2.32 | 0.86 | 0.781 |
|  | female | 0.69 | 1.06 | 0.09 |  |  | 0.93 | 1.77 | 0.82 |  |
| Stage | Stage II | 0.95 | 1.75 | 0.86 | 0.656 |  | 0.91 | 2.43 | 0.85 | 0.822 |
|  | Stage III | 0.81 | 1.20 | 0.29 |  |  | 1.03 | 1.82 | 0.93 |  |
| Smoking | never | 0.83 | 1.26 | 0.38 | 0.791 |  | 1.12 | 2.13 | 0.74 | 0.482 |
|  | former | 0.91 | 1.57 | 0.74 |  |  | 0.77 | 1.67 | 0.51 |  |
| ECOG PS | 0 | 0.74 | 1.08 | 0.12 | 0.077 |  | 0.77 | 1.36 | 0.37 | 0.064 |
|  | 1 | 1.61 | 3.32 | 0.19 |  |  | 2.46 | 7.34 | 0.11 |  |
| TMB (median) | high | 1.01 | 0.55 | 1.84 | 0.786 |  | 0.89 | 0.35 | 2.23 | 0.906 |
|  | low | 0.93 | 0.53 | 1.66 |  |  | 0.97 | 0.39 | 2.41 |  |
| *EGFR* mutation | ex19 del | 0.97 | 0.55 | 1.72 | 0.744 |  | 0.81 | 0.34 | 1.94 | 0.511 |
|  | L858R | 0.86 | 0.51 | 1.46 |  |  | 1.24 | 0.57 | 2.67 |  |
| *TP53* | mutant | 0.80 | 0.48 | 1.34 | 0.578 |  | 0.70 | 0.32 | 1.50 | 0.143 |
|  | wt | 0.98 | 0.55 | 1.75 |  |  | 1.70 | 0.68 | 4.27 |  |
| *CSMD3* | mutant | 1.73 | 0.57 | 5.21 | 0.168 |  | 1.38 | 0.19 | 10.22 | 0.620 |
|  | wt | 0.82 | 0.55 | 1.24 |  |  | 0.95 | 0.52 | 1.72 |  |
| *NOTCH1* | mutant | 1.40 | 0.42 | 4.62 | 0.401 |  | 5.49 | 1.08 | 28.00 | **0.039*** |
|  | wt | 0.84 | 0.56 | 1.26 |  |  | 0.79 | 0.42 | 1.49 |  |
| *SYNE1* | mutant | 0.66 | 0.20 | 2.19 | 0.651 |  | 0.44 | 0.07 | 2.65 | 0.304 |
|  | wt | 0.91 | 0.61 | 1.36 |  |  | 1.13 | 0.62 | 2.07 |  |
| *KMT2C* | mutant | 2.29 | 0.54 | 9.70 | 0.103 |  | 4.21 | 0.49 | 36.16 | 0.147 |
|  | wt | 0.81 | 0.54 | 1.20 |  |  | 0.85 | 0.46 | 1.57 |  |
| *KMT2D* | mutant | 1.42 | 0.32 | 6.40 | 0.452 |  | 0.88 | 0.19 | 4.13 | 0.860 |
|  | wt | 0.83 | 0.56 | 1.23 |  |  | 1.05 | 0.56 | 1.95 |  |
| *LRP1B* | mutant | 0.84 | 0.24 | 2.97 | 0.919 |  | 0.84 | 0.12 | 6.09 | 0.904 |
|  | wt | 0.89 | 0.59 | 1.32 |  |  | 1.02 | 0.56 | 1.86 |  |
| *PIK3CA* | mutant | 1.03 | 0.24 | 4.37 | 0.933 |  | 1.62 | 0.10 | 25.96 | 0.759 |
|  | wt | 0.89 | 0.60 | 1.32 |  |  | 0.98 | 0.54 | 1.75 |  |
| *RB1* | mutant | 0.33 | 0.06 | 1.68 | 0.099 |  | 0.26 | 0.02 | 2.88 | 0.221 |
|  | wt | 0.98 | 0.66 | 1.44 |  |  | 1.13 | 0.62 | 2.04 |  |
| *STK11* | mutant | 0.51 | 0.10 | 2.59 | 0.469 |  | NE | NE | NE | 0.999 |
|  | wt | 0.93 | 0.63 | 1.37 |  |  | 1.14 | 0.64 | 2.05 |  |
| *USP9X* | mutant | 0.67 | 0.16 | 2.86 | 0.960 |  | 1.34 | 0.22 | 8.18 | 0.761 |
|  | wt | 0.90 | 0.61 | 1.34 |  |  | 1.02 | 0.55 | 1.86 |  |
| *APC* | mutant | 0.50 | 0.12 | 2.01 | 0.351 |  | 0.72 | 0.12 | 4.49 | 0.832 |
|  | wt | 0.94 | 0.63 | 1.40 |  |  | 1.05 | 0.57 | 1.92 |  |
| *TAF1* | mutant | 1.21 | 0.23 | 6.28 | 0.902 |  | 0.69 | 0.06 | 7.69 | 0.887 |
|  | wt | 0.87 | 0.59 | 1.29 |  |  | 0.95 | 0.52 | 1.74 |  |
| *SMO* | mutant | 0.33 | 0.03 | 3.15 | 0.316 |  | NE | NE | NE | 0.999 |
|  | wt | 0.92 | 0.63 | 1.36 |  |  | 0.96 | 0.54 | 1.71 |  |
| *DCC* | mutant | 2.17 | 0.43 | 11.06 | 0.294 |  | NE | NE | NE | 0.999 |
|  | wt | 0.86 | 0.58 | 1.28 |  |  | 0.86 | 0.48 | 1.56 |  |
| *TRRAP* | mutant | 0.21 | 0.02 | 1.82 | 0.163 |  | NE | NE | NE | 0.232 |
|  | wt | 0.95 | 0.64 | 1.41 |  |  | 1.13 | 0.62 | 2.05 |  |
| *BRD3* | mutant | 0.97 | 0.09 | 10.84 | 0.926 |  | 1.58 | 0.09 | 27.19 | 0.600 |
|  | wt | 0.87 | 0.59 | 1.27 |  |  | 0.98 | 0.55 | 1.77 |  |
| *CREBBP* | mutant | NE | NE | NE | **0.058*** |  | 0.23 | 0.02 | 2.45 | 0.257 |
|  | wt | 0.97 | 0.65 | 1.44 |  |  | 1.13 | 0.62 | 2.05 |  |
| *IGF2R* | mutant | 1.78 | 0.18 | 17.23 | 0.303 |  | NE | NE | NE | 0.999 |
|  | wt | 0.87 | 0.59 | 1.28 |  |  | 1.12 | 0.62 | 2.03 |  |
| *RET* | mutant | 1.37 | 0.26 | 7.09 | 0.613 |  | 2.21 | 0.20 | 24.47 | 0.517 |
|  | wt | 0.87 | 0.59 | 1.29 |  |  | 0.97 | 0.53 | 1.74 |  |

Subgroup analyses of disease-free survival and overall survival between gefitinib and cis/vin groups and interaction tests. The interaction of each biomarker was tested using the Cox proportional hazards model and P for interaction was calculated, and a P-value of < 0.1 indicates a possible interaction between treatment and marker. Abbreviations: ECOG, Eastern Cooperative Oncology Group; PS, Performance Status; TMB, tumor mutation burden; TP53, Tumor Protein p53; CSMD3, CUB and Sushi multiple domains 3; SYNE1, Spectrin Repeat Containing Nuclear Envelope Protein 1; KMT, Lysine (K)-specific methyltransferase; LRP1B, LDL Receptor Related Protein 1B; PIK3CA, Phosphatidylinositol-4,5-bisphosphate 3-kinase catalytic subunit alpha; RB1, RB Transcriptional Corepressor 1; STK11, serine/threonine kinase 11; USP9X, Ubiquitin Specific Peptidase 9 X-Linked; APC, adenomatous polyposis coli; TAF1, TATA-Box Binding Protein Associated Factor 1; SMO, Smoothened, Frizzled Class Receptor; DCC, deleted in colorectal cancer; TRRAP, Transformation/Transcription Domain-Associated Protein; BRD3, Bromodomain Containing 3; CREBBP, cAMP response element binding protein; IGF2R, Insulin Like Growth Factor 2 Receptor; RET, ret proto-oncogene.
